# Therapeutic validity and replicability of power training interventions in older adults: A review using the TIDieR checklist and CONTENT scale

**DOI:** 10.1016/j.heliyon.2024.e24362

**Published:** 2024-01-12

**Authors:** Mohamed el Hadouchi, Henri Kiers, Brittany A. Boerstra, Cindy Veenhof, Jaap van Dieën

**Affiliations:** a Institute for Human Movement Studies, University of Applied Sciences Utrecht, Heidelberglaan 7, 3584, CS, Utrecht, the Netherlands; b Department of Human Movement Sciences, Vrije Universiteit Amsterdam, Amsterdam Movement Sciences, van der Boechorststraat 7, 1081, BT, Amsterdam, the Netherlands; c HRC, Sports & Science Health, Hoeflingweg 20, 7241, CH, Lochem, the Netherlands; d Research Group Innovation of Movement Care, University of Applied Sciences Utrecht, Heidelberglaan 7, 3584, CS, Utrecht, the Netherlands; e Department of Rehabilitation, Physical Therapy Science and Sport, Brain Center, University Medical Center Utrecht, Heidelberglaan 100, 3584, CX, Utrecht, the Netherlands

**Keywords:** Power training, Replicability, Validity, Older adults

## Abstract

**Background:**

Randomized controlled trials (RCTs) indicate that power training has the ability to improve muscle power and physical performance in older adults. However, power training definitions are broad and previously-established criteria are vague, making the validity and replicability of power training interventions used in RCTs uncertain.

**Objective:**

The aim of this review was to assess whether the power training interventions identified in a previous systematic review (el Hadouchi 2022) are fully described, therapeutically valid, and meet our proposed criteria for power training.

**Design:**

Review.

**Methods:**

Power training interventions used in older adults, previously-identified in a systematic review, were assessed. The completeness of intervention descriptions was evaluated using the Template for Intervention Description and Replication (TIDieR), and therapeutic validity was evaluated using the CONTENT scale in combination with a set of criteria specific for power training.

**Results:**

None of the power training interventions were fully described or met the CONTENT scale's criteria for therapeutic validity. Five out of 14 interventions (35.7 %) met all specific power training criteria.

**Conclusions:**

Power training interventions used in RCTs comparing power training to strength training are poor to moderately described, may not be therapeutically valid, and may not reflect the construct of power training. This makes it difficult for clinicians or researchers to apply or replicate power training interventions reported in RCTs, and begs the question whether the true effects of power training have been estimated.

## Highlights

• Randomized controlled trials poorly describe power training used in older adults.
• Power training interventions may not be therapeutically valid.
• Interventions may not reflect the construct of power training.

## 1 Introduction

Research suggests that power training offers more potential for improving physical performance and functioning in older adults compared to strength training alone [[1], [2], [3], [4], [5]]. Power training is a dynamic exercise training that requires a higher velocity of muscular contractions compared to traditional strength training and emphasizes the development of type 2 muscle fibers responsible for shorter bursts of explosive movement [6,7]. These type 2 muscle fibers, also known as fast twitch fibers, deteriorate at a faster rate with older age, thereby reducing one's ability to generate muscle strength rapidly [7].

The age-related decline in muscular condition has been expressed in terms of loss of muscle strength (the ability to produce large muscle force) and muscle power (the ability to produce a large muscle force at high contraction velocity) [4,8,9]. Several studies revealed that in older adults, the annual decline in muscle power is larger than the annual decline in muscle strength [2,[10], [11], [12], [13], [14], [15]], making the ability to move with sufficient velocity (emphasizing muscle power) more often the limiting factor than the ability to produce sufficient muscle force (emphasizing muscle strength) [3,10]. As a result, aging is often accompanied by functional limitations, increased risk of falls, reduced movability, and a decreased quality of life [16].

Power training has been shown to increase muscle power even in older adults [3,17]. However, the descriptions of power training interventions used in randomized controlled trials (RCTs) are often incomplete and lack clarity, hindering replicability. Additionally, power training definitions used in RCTs are vague and there is a lack of consensus regarding the elements that need to be fulfilled for an exercise intervention to constitute as power training. As a result, there are large differences in applied power training interventions [5]. The heterogeneity between power training interventions raises the question whether the interventions used in RCTs adequately target muscle power and are therapeutically valid.

Therapeutic validity has been defined as the potential effectiveness of a specific intervention given the target group [18]. Only with a clear description of the intervention is available, it is possible to determine whether the intervention is potentially effective, in this case whether the intervention meets the definition of power training and is suitable for older adults. At this moment, there is no clear and comprehensive framework that specifies the essential components of power training, contributing to the frequently encountered confusion between power training and strength training. Therefore, we developed a set of power training criteria based on biomechanical principles and exercise physiology theories proposed by Kraemer et al. [6] and Haff et al. [19] to assess to which extent the interventions used in RCTs reflected power training principles. These power training criteria evaluate: (1) type of exercises; (2) movement pace; (3) rate of force development; (4) training load; and (5) duration of the intervention.

In this review, we aimed to assess whether the power training interventions identified in a previous systematic review by el Hadouchi et al. [5] are fully described, therapeutically valid, and meet our proposed criteria for power training. Answering these questions will provide insight into whether the conclusions drawn from the included RCTs evaluating the effects of power training interventions in older adults were warranted. Furthermore, addressing these gaps in the literature will provide a framework for the necessary improvements for future research reporting on power training interventions or other exercise interventions.

## 2 Methods

Power training interventions were rated on whether they were described in sufficient detail, to what extent they showed therapeutic validity, and whether they met our proposed criteria for power training. The scope of this review is limited to RCTs included in an existing systematic review comparing the effectiveness of power training to strength training in older adults [5], but the search was updated for the present review. This review was registered in the International Prospective Register of Systematic Reviews (PROSPERO 2021: CRD42021273832).

### 2.1 Search strategy and study selection

**Fig. 1:**
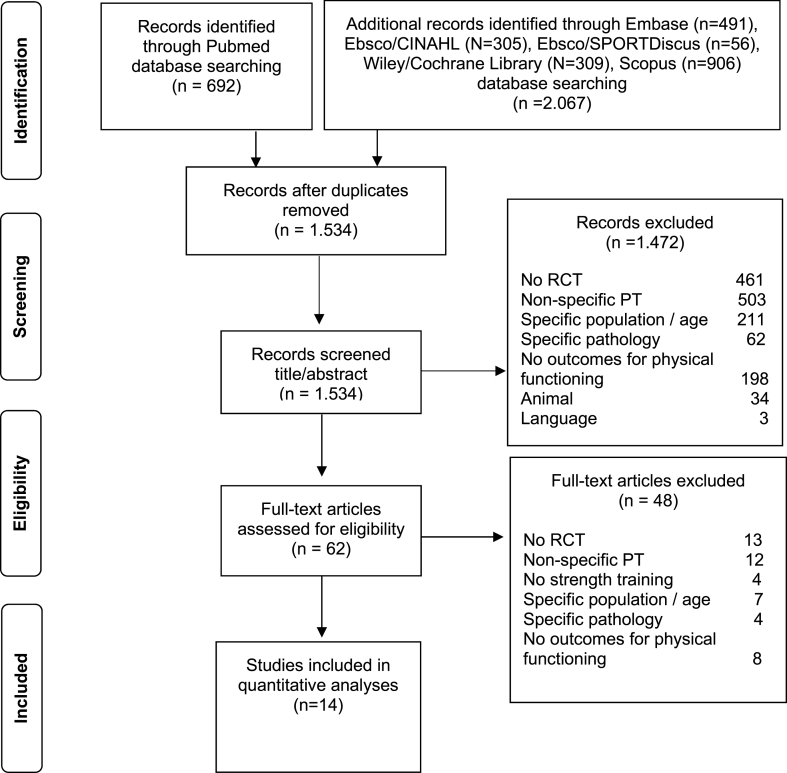
PRISMA flow diagram of literature search and study selection. Abbreviations used: RCT: randomized controlled trial; PT: power training.

The original systematic search was performed in PubMed, Embase, Ebsco/CINAHL, Ebsco/SPORTDiscus, Wiley/Cochrane Library and Scopus up until September 18, 2020 in collaboration with a medical librarian. The search string (Appendix 1) was applied once more to all abovementioned databases on August 7, 2023 to search for any more recently-published literature. Study selection was performed independently by two coauthors (MeH and HK) by first selecting relevant titles and abstracts using the online software Rayyan [20], followed by a full-text screening. RCTs that compared a power training intervention to a strength training intervention in older adults were included if: (a) the mean age of the study population was >65 years and participants were recruited from a ‘healthy'population. Healthy was defined using the WHO definition for health, in which individuals can be considered healthy despite the presence of (chronic) health conditions; (b) the intervention was defined as power training by the authors or the intervention met the definition of power training proposed by Haff et al. [19]: “an intervention primarily aimed at muscle power, movement speed or rate of force development”; (c) the study included outcome measures for muscle power, activity-based tests, or physical functioning in daily life; (d) the strength training control group was age-matched and received at least partially supervised strength training; and (e) studies were written in English, Dutch, or German. Studies were excluded if the study population consisted of solely of participants with specific (medical) conditions on the basis on non-generalizability. Furthermore, studies were excluded if the interventions were home- or internet-base in view of concerns regarding adherence. A detailed explanation of the search strategy, study selection, and risk of bias assessment can be found in the primary study [5]. Fig. 1 summarizes the literature search and study selection using a PRISMA flow diagram [21].

### 2.2 Completeness of descriptions

The completeness of interventions descriptions was assessed using the Template for Intervention Description and Replication Checklist (TIDieR) checklist [22], which consists of 12 items that assess whether interventions are described in sufficient detail. The goal of the TIDieR checklist is to assess whether interventions are described in sufficient detail to allow their replication or translation into clinical practice. The TIDieR checklist was applied independently by two researchers (MeH and BB) with regular meetings to form consensus. If no consensus was reached, a third researcher was consulted and a consensus was reached through discussion between all three.

Within the TIDieR checklist, criterion 8 aims to identify the number of training sessions, their schedule, duration, intensity, and dose. We felt that additional information was needed to further specify relevant details. Therefore, we expanded criterion 8 to include the following training parameters: a) total number of sessions, b) frequency (trainings/week), c) duration of a session, d) intensity, e) type of exercises, f) level of exercise (the degree of difficulty of the exercises), and g) combination with other exercises or interventions. The expansion of this criterion was done in consultation with professionals (researchers, sports and geriatric physiotherapists, and strength and conditioning trainers) by asking them which training parameters are of importance when evaluating and reproducing an exercise intervention. Each item on the TIDieR checklist was scored with no (0) or yes (1). For criterion 8, a point was only awarded if all training parameters were described. In the absence of a scoring guideline, we used the following interpretation of the scores: 0–4 points were considered poor, 5–9 points were considered moderate, and 10–12 points was considered good. The scoring system emphasizes the completeness and transparency of what has been reported rather than a qualitative ranking of interventions.

### 2.3 Therapeutic validity

Therapeutic validity was defined as “the potential effectiveness of a specific intervention given the target group” and was assessed using the validated CONTENT scale for the therapeutic validity of therapeutic exercise programs [18]. The CONTENT scale was completed independently by two researchers (MeH and BB), after which consensus was formed. If no consensus was reached, a third researcher was consulted and a consensus was reached through discussion between the three. The checklist consists of 9 criteria that, if described and met, are worth 1 point each. An intervention was considered therapeutically valid if the total score was >6 points.

### 2.4 Power training criteria

Item 5 of the CONTENT scale includes the question:“Was the rationale for the content and intensity of the therapeutic practice described and plausible?“. Due to the importance of this item in determining whether the exercises used are consistent with the principles of power training, we developed a specific set of criteria to assess to what extent the interventions used in RCTs reflect power training principles. These power training criteria are based on biomechanical principles and exercise physiology theories proposed by Kraemer et al. [6] and Haff et al. [19] and are listed in Table 1. The results of item 5 are interpreted separate from the CONTENT scale.

**Table 1:** Power training criteria.

#	Concept	Criteria
1	Type of exercises	The training consists of exercises that emphasize the speed of the performance (e.g. Olympic weightlifting or strength exercises that have been adapted into power exercises).
2	Movement pace	The instruction and execution of the power exercises is “as fast as possible”.
3	Rate of force development	Each exercise should be characterized by a fast and powerful concentric phase, followed by a slow and steady eccentric phase.
4	Training load	The resistance of the exercises is low. The intervention uses a training load of 20–30 % of the 1-repetition maximum (1RM) OR, a build-up range is used between 0 and 60 % 1RM (0–60 % 1RM for lower extremity exercises and 30–60 % 1RM for upper extremity exercises).
5	Duration	The minimum duration of the intervention is 8 weeks with a training frequency of at least twice a week.

## 3 Results

### 3.1 Study characteristics

The updated search yielded 21 additional recently-published studies, none of which met eligibility criteria. Therefore, a total of 14 power training interventions from previously-identified RCTs were included. Table 2 provides an overview of the study characteristics and training parameters. The RCTs varied greatly with regard to the setting, the supervision, the length of follow-up, and the training parameters. A majority of power training interventions utilized exercise machines (53 %), while the remaining interventions used own body weight exercises, task-specific exercises such as step up and step down to simulate stair climbing (7 %), or a combination of both (40 %). The power training interventions were supervised by exercise trainers (60 %) or student physicians (20 %). In the remaining 20 % of studies, the level of supervision was unclear. Twenty percent of training sessions were performed in a gym, 20 % in an exercise room of a clinical setting, and 13 % at a rehabilitation center. The age of the participants ranged from 66 to 93 years (median 72 years), with a large majority of participants considered “community-dwellers” who lived independently or in a nursing home.

**Table 2:** Study characteristics and training parameters of the included studies.

Study (year)	Type of training	Total sessions	Frequency (sessions/week)	Duration (min)	Intensity	Type of exercises	# sets	# reps	Rest in between sets (min)	Movement speed	Supervision	Tailoring
Balachandran (2014) [16]	PT	30	2	40–45	50–80 % 1RM	Machines	3	10–12	2	CON: HS; ECC: LS	Exercise trainer	5 % increase in load once participant could perform 3 × 12 reps
Bean (2009) [4]	PT	48	3	40–60	11-16 RPE	Body and task-specific exercises	2	10	1	CON: HS; ECC: LS	Exercise trainer	Decrease in load at RPE >17; decrease in load at RPE <11
Bottaro (2007) [23]	PT	20	2	NR	40–60 % 1RM	Machines	3	8–10	NR	CON: HS; ECC: NS	NR	NR
Fielding (2002) [24]	PT	48	3	NR	70 % 1RM	Machines	3	8–10	NR	CON: HS; ECC: NS	Exercise trainer	Biweekly 1RM measurement to ensure that exercise intensity remained at 70 % 1RM
Henwood (2006) [25]	HVT	16	2	60	45–70 % 1RM	Combination of machines and body- and task-specific exercises	3	10	1	CON: HS; ECC: NS	Exercise trainer	5–10 % increase in load at >10 reps
Henwood (2008) [26]	PT	48	2	60	45–75 % 1RM	Machines	3	8	1	CON: HS; ECC: LS	Exercise trainer	5–10 % increase in load at >10 reps
Lopes (2014) [27]	PT	36	3	NR	30–50 % 1RM	Machines and free weights	3	2–7	NR	CON: HS; ECC: NS	NR	Biweekly 1RM measurement to ensure exercise intensity remained between 30 and 50 % 1RM
Marsh (2009) [28]	PT	36	3	40	70 % 1RM	Machines	3	8–10	NR	CON: HS; ECC: LS	ACSM-certified interventionists	Biweekly 1RM measurement to ensure that exercise intensity remained at 70 % 1RM
Miszko (2003) [17]	PT	48	3	NR	40 % 1RM	Combination of machines and body- and task-specific exercises	3	6–8	NR	CON: HS; ECC: LS	NR	NR
Orr (2006) [29]	PT	20	2	NR	20 %, 50 %, and 80 % 1RM	Machines	3	8	NR	CON: HS; ECC: LS	Exercise trainer	Weekly 1RM measurement to ensure that exercise intensity remained at target intensity
Ramirez-Campillo (2014) [30]	HVT	36	3	70	45–75 % 1RM	Machines and free weights	3	8	1	CON: HS; ECC: LS	Exercise trainer	Increase in load at >8 reps
Reid (2015) [31]	PT	32	2	NR	40, 70 % 1RM	Machines	3	8–10	NR	CON: HS; ECC: NS	Other	NR
Tiggeman (2016) [32]	PT	24	2	NR	13-18 RPE/45–65 % 1RM	Machines	2	8–10	2	CON: HS; ECC: LS	Other	Decrease in load at RPE >18; increase in load at RPE <13
Zech (2012) [33]	PT	24	2	NR	10-16 RPE	Combination of machines, body- and task-specific exercises, and balance	2	NR	2	CON: HS; ECC: LS	Other	Biweekly increase to 16 RPE

Abbreviations use: PT: power training; HVT: high-velocity training; 1RM: 1-repetition maximum; RPE: rate of perceived exertion; NR: not reported; CON: concentric: ECC: eccentric; HS: high-speed; NS: normal-speed; LS: low-speed.

### 3.2 Completeness of descriptions

None of the RCTs described all the items on the TIDieR checklist. The quality of reporting the featured power training interventions was scored poor to moderate (Table 3). Twelve of the RCTs described the procedures, activities, and/or processes used in the power training intervention (criterion 4), but only 7 of RCTs described a rationale for the intervention (criterion 2). Wth respect to criterion 8, all RCT's described at least one training parameter (criteria 8a-g), but none of the RCTs described all parameters (mean: 5, range: 3–6). Most notably, criterion 10 for ‘modification’, criterion 11 for ‘adherence’; and criterion 12 for ‘deliverance’ were not reported in any of the RCTs. A complete overview of findings related to the TIDieR checklist can be found in **S1 Table.**

**Table 3:** Overview of findings.

Tool	Criteria	Balachandran (2014)	Bean (2009)	Bottaro (2007)	Fielding (2002)	Henwood (2006)	Henwood (2008)	Lopes (2014)	Marsch (2009)	Miszko (2003)	Orr (2006)	Ramirez-Campillo (2014)	Reid (2013)	Tiggeman (2016)	Zech (2012)	Frequency of reporting
TIDieR	Total score	7	6	3	7	7	8	4	7	4	7	5	4	8	8	
CONTENT scale	Total score	5	5	4	5	5	5	5	5	4	5	5	5	5	4	
Power training criteria	Type of exercises	✓	-	✓	✓	✓	✓	✓	✓	✓	✓	✓	✓	✓	✓	93%
	Movement pace	✓	✓	✓	✓	✓	✓	✓	✓	✓	✓	✓	✓	✓	✓	100%
	Rate of force development	✓	✓	✓	✓	✓	✓	✓	✓	✓	✓	✓	✓	✓	✓	100%
	Training load	-	-	✓	-	-	-	✓	-	✓	✓	-	✓	-	-	36%
	Duration	✓	✓	✓	✓	✓	✓	✓	✓	✓	✓	✓	✓	✓	✓	100%

✓=reported; - = not reported. The following score system was used to determine replicability of studies as determined through the TIDieR checklist: 0–4 poor; 5–9 moderate; 10–12 points good. Interventions with a CONTENT score >6 points are considered therapeutically valid. Abbreviations use: PT: power training

### 3.3 Therapeutic validity

None of the power training interventions were considered therapeutically valid (>6 points), as all of the power training intervention scored between 4 and 5 points on the CONTENT scale. Criteria 1 and 2 regarding patient selection, criterion 4 evaluating whether the intervention was ‘based on a priori aims and intentions’, and criterion 6 evaluating whether ‘the intensity of the intervention was described’ were met by all studies. Criterion 7 for ‘monitoring and adjustment of intervention’ was met by 11 of the 14 studies. There were also several criteria that were met by only a few of the selected studies. Criterion 3 for ‘eligibility criteria for therapist and setting’, criterion 5 for ‘rationale for content and intensity of intervention’; criterion 8 for whether the intervention was ‘personalized and contextualized for individual participants’, and criterion 9 for ‘adherence determined and acceptable’ were scored as insufficient for all of the studies. In applying criterion 5 for ‘rationale for content and intensity of therapeutic exercise’ it became clear that most studies did not indicate the rationale for the chosen intensity or content of the intervention. The training intensity varied widely between studies. Two studies used the Borg-scale rate of perceived exertion (RPE) to describe training intensity, while the remaining studies used a range of 1RM percentages to describe the training intensity. The rationale for the content and intensity of therapeutic exercise was lacking in all of the studies. A complete list of therapeutic validity scores can be found in S2 Table.

### 3.4 Power training criteria

Power training criteria were applied to each of the 14 interventions. The criterion for type of exercises was met by 13 out of 14 interventions, 1 study did not specify the exercises used. The criterion for movement pace and rate of force development were met by all interventions. The criterion for training load was met by only 5 interventions, because a majority of interventions used higher training loads, or determined training load through a rating of perceived exertion (RPE). Lastly, the criterion for duration was also met by all interventions. A total of 5 interventions (36 %) met all power training criteria.

## 4 Discussion

Our results show that the power training interventions used in older adults were poor to moderately described, as they did not meet the criteria for replicability established by the TIDieR checklist. Most notably, information regarding the modification, adherence, and deliverance of the power training intervention was not provided in any of the included RCTs. Furthermore, none of the power training interventions were considered therapeutically valid and only a third of interventions met the proposed power training criteria.

To our best knowledge, this is the first study to evaluate power training interventions featured in RCTs using the TIDieR checklist and CONTENT scale. There are several systematic reviews evaluating the effects of power training in older adults [[34], [35], [36]], even comparing power training to strength training [[37], [38], [39]], but the content of power training interventions was not evaluated in these studies. Previous reviews also noted the heterogeneity in findings between RCTs evaluating the effects of power training in older adults, which could be the result of differences in power training protocols.

Differences in training protocols, in addition to differences in outcome measurement, can greatly increase the heterogeneity of findings across studies even if these have a similar methodology. This heterogeneity increases uncertainty about the effectiveness of power training. Additionally, there is a lack of consensus with regard to the ideal training load for power training interventions, which hampers the implementation of power training interventions in clinical practice. The relationship between muscle force and contraction velocity is such that as the magnitude of force required to move an external object increases, the velocity at which a muscle is capable of moving the external object decreases. This inverse relationship gives rise to two distinct approaches for training muscle power: (1) the high force and low velocity approach (∼70 % 1RM) and, (2) the low force and high velocity approach (∼30 % 1RM). Direct comparisons indicate that interventions using a training load between 20 and 30 % 1RM were most effective at improving muscle power in older adults [6,7,16,19,40,41], but there are still studies that use power training interventions with a higher load. However, more generally the lack of clarity in the description of training interventions investigated precludes determination of which intervention characteristics s contribute most to effectiveness of power training. This not only hinders the understanding of the effectiveness of power training in older adults, but also hampers the implementation of power training interventions.

Standardizing the way in which RCTs report on power training interventions and other exercise interventions could improve the assessment of therapeutic validity, increase replication of results, and promote implementation. From the RCTs included in the present review that did not describe the intervention fully, it could not be determined whether the criteria from the CONTENT scale for therapeutic validity were considered but not reported, or not considered at all. More specifically, missing information with regards to the reporting of modification, adherence, and deliverance of power training interventions indicate a lack of quality in these areas.

We propose that future research should use the TIDieR checklist in combination with the CONSORT checklists and CONTENT scale to ensure that a complete description of the intervention is reported and that the content of the intervention reflects the construct of power training. While this may increase the word counts of papers reporting RCTs, we believe that adherence to these checklists on an a priori basis can improve the interpretations, assessment, and understanding of the effects of power training in older adults.

Certain criteria within the TIDieR checklist and CONTENT scale were considered to be more relevant to the construct of power training than others. Specifically, criterion 8 of the TIDieR checklist was expanded to include power training parameters not fully covered by the original checklist. Yet, this criterion was not fully met by any of the included RCTs. Furthermore, criterion 2 of the CONTENT scale, which evaluates the presence of a rationale, was met by only half of the included studies. Although a rationale is not necessary to replicate the intervention, a hypothesis about the mechanism by which the intervention has an effect should be established prior to testing the effectiveness of the intervention to increase therapeutic validity and justify the use of appropriate measurement tools.

There are several limitations to this review. Because this review is a secondary analysis and used the same search strategy as el Hadouchi et al. [5], the scope of the review is limited to evaluating interventions used in RCTs comparing the effects of power training to strength training in older adults with muscle power, activity-based tests, and physical activity level in daily life as outcomes. Additionally, because the CONTENT scale assess the therapeutic validity of power training interventions based on how well and complete the interventions were described, it is possible that the intervention was indeed power training and performed well, but was not adequately described in the article. Lastly, the dichotomous response options in the TIDieR checklist limit the interpretation of these criteria and an ordinal or qualitative response would likely have been more informative. To compensate for this, we provided a thorough description of these criteria in Table 2. Further strengths of this review include the incorporation of power training criteria to expand the CONTENT scale's ability to evaluate the intensity and type of exercises used in the power training interventions. While these proposed power training criteria were developed by experts, it would be advisable to get broader consensus whether these criteria accurately represent the construct of power training.

## 5 Conclusions

Power training interventions used in RCTs comparing power training to strength training are poor to moderately described, may not be therapeutically valid, and may not reflect the construct of power training. These factors make it difficult for clinicians or researchers to interpret, translate or replicate power training interventions reported in RCTs, and beg the question whether the true effects of power training have been estimated. Future RCTs evaluating the effects of power training in older adults should guarantee therapeutic validity, and provide clearer and more complete descriptions of the intervention.

## Funding

This work was supported by the 10.13039/501100003246Dutch Research Council (NWO 2017/BOO/00279639). The funding source played no role in the development or conducting of the study.

## Data availability statement

No data was used for the research described in this article.

## CRediT authorship contribution statement

**Mohamed el Hadouchi:** Writing - original draft, Visualization, Project administration, Methodology, Formal analysis, Data curation, Conceptualization. **Henri Kiers:** Writing - review & editing, Supervision, Methodology, Funding acquisition, Conceptualization. **Brittany A. Boerstra:** Writing - review & editing, Visualization, Formal analysis. **Cindy Veenhof:** Writing - review & editing, Supervision, Methodology, Funding acquisition, Conceptualization. **Jaap van Dieën:** Writing - review & editing, Supervision, Methodology, Funding acquisition, Conceptualization.

## Declaration of competing interest

The authors declare that they have no known competing financial interests or personal relationships that could have appeared to influence the work reported in this paper.
