# Supplementary material for: Therapeutic validity and replicability of power training interventions in older adults: A review using the TIDieR checklist and CONTENT scale
Source: Heliyon. 2024 Jan 12;10(2):e24362. doi: 10.1016/j.heliyon.2024.e24362 (PMC10827759; doi:10.1016/j.heliyon.2024.e24362)
Supplement: Multimedia component 1 [file mmc1.docx]

**S1. PubMed search string used in literature search.**

(("Aged"[Mesh] OR "Aging"[Mesh] OR "Homes for the Aged"[Mesh] OR "Housing for the Elderly"[Mesh] OR "Geriatrics"[Mesh] OR "Geriatric Nursing"[Mesh] OR "Health Services for the Aged"[Mesh] OR (elder*[tw] OR eldest[tw] OR frail*[tw] OR geriatri*[tw] OR old age*[tw] OR oldest old*[tw] OR senior*[tw] OR senium[tw] OR very old*[tw] OR septuagenarian*[tw] OR octagenarian*[tw] OR octogenarian*[tw] OR nonagenarian*[tw] OR centarian*[tw] OR centenarian*[tw] OR supercentenarian*[tw] OR older people[tw] OR older subject*[tw] OR older patient*[tw] OR older age*[tw] OR older adult*[tw] OR older man[tw] OR older men[tw] OR older male*[tw] OR older woman[tw] OR older women[tw] OR older female*[tw] OR older population*[tw] OR older person*[tw]) OR aging[tiab] OR ageing[tiab] OR community-dwell*[tiab]) AND ("Self Efficacy"[Mesh] OR "Activities of Daily Living"[Mesh] OR "Quality of Life"[Mesh] OR self efficac*[tiab] OR "activities of daily living"[tiab] OR "activity of daily living"[tiab] OR "activities of daily life"[tiab] OR "activity of daily life"[tiab] OR daily living activit*[tiab] OR daily life activit*[tiab] OR adl[tiab] OR iadl[tiab] OR "quality of life"[tiab] OR life qualit*[tiab] OR living qualit*[tiab] OR "quality of living"[tiab] OR "qol"[tiab] OR "hrql"[tiab] OR "hrqol"[tiab] OR functional abilit*[tiab] OR physical abilit*[tiab] OR functional perform*[tiab] OR physical perform*[tiab] OR functional independen*[tiab] OR physical independen*[tiab] OR "Personal Autonomy"[Mesh] OR functional autonom*[tiab] OR physical autonom*[tiab] OR "Physical Fitness"[Mesh] OR Independent living[tiab] OR Living independent*[tiab] OR high function*[tiab] OR higher function*[tiab] OR physical function*[tiab]) AND ((power[tiab] OR plyometric*[tiab] OR high velocit*[tiab] OR high intens*[tiab] OR explosive strength[tiab]) AND ("Exercise"[Mesh] OR "Sports"[Mesh] OR "Exercise Therapy"[Mesh] OR training[tiab] OR physical activity[tiab] OR exercise[tiab]))) AND (randomized controlled trial[pt] OR controlled clinical trial[pt] OR randomized[tiab] OR randomised[tiab] OR placebo[tiab] OR randomly[tiab] OR trial[tiab] OR groups[tiab])

**S2 Table.** TIDier checklist for replicability

|  |  |  | | Balachandran (2014) | | Bean (2009) | | Bottaro (2007) | | Fielding (2002) | | Henwood (2006) | | Henwood (2008) | | Lopes (2014) | | Marsch (2009) | | Miszko (2003) | | Orr (2006) | | Ramirez-Campillo (2014) | | Reid (2013) | | Tiggeman (2016) | | Zech (2012) | | Frequency of reporting | |
| --- | --- | --- | --- | --- | --- | --- | --- | --- | --- | --- | --- | --- | --- | --- | --- | --- | --- | --- | --- | --- | --- | --- | --- | --- | --- | --- | --- | --- | --- | --- | --- | --- | --- |
| 1 | Intervention | | 1 | | 1 | | 1 | | 1 | | 1 | | 1 | | 1 | | 1 | | 1 | | 1 | | 1 | | 1 | | 1 | | 1 | | 100% | |  |
| 2 | Rationale | | 0 | | 1 | | 1 | | 0 | | 1 | | 1 | | 0 | | 0 | | 0 | | 1 | | 0 | | 0 | | 1 | | 1 | | 50% | |  |
| 3 | Materials | | 1 | | 1 | | 0 | | 1 | | 1 | | 1 | | 1 | | 1 | | 1 | | 1 | | 0 | | 0 | | 1 | | 1 | | 80% | |  |
| 4 | Procedure | | 1 | | 1 | | 1 | | 1 | | 1 | | 1 | | 1 | | 1 | | 1 | | 1 | | 0 | | 0 | | 1 | | 1 | | 85.7% | |  |
| 5 | Person | | 1 | | 1 | | 0 | | 1 | | 1 | | 1 | | 0 | | 1 | | 0 | | 1 | | 1 | | 1 | | 1 | | 1 | | 78.6% | |  |
| 6 | Modes | | 1 | | 1 | | 0 | | 1 | | 1 | | 1 | | 0 | | 1 | | 1 | | 1 | | 1 | | 1 | | 1 | | 1 | | 85.7% | |  |
| 7 | Location | | 1 | | 0 | | 0 | | 1 | | 1 | | 1 | | 0 | | 1 | | 0 | | 0 | | 0 | | 0 | | 1 | | 1 | | 50% | |  |
| 8 | Training parameters | | 0 | | 0 | | 0 | | 0 | | 0 | | 0 | | 0 | | 0 | | 0 | | 0 | | 0 | | 0 | | 0 | | 0 | | 0% | |  |
|  | *a) Total number of sessions* | | 🗸 | | 🗸 | | 🗸 | | 🗸 | | 🗸 | | 🗸 | | 🗸 | | 🗸 | | 🗸 | | 🗸 | | 🗸 | | 🗸 | | 🗸 | | 🗸 | | *100%* | |  |
|  | *b) Frequency (sessions/week)* | | 🗸 | | 🗸 | | 🗸 | | 🗸 | | 🗸 | | 🗸 | | 🗸 | | 🗸 | | 🗸 | | 🗸 | | 🗸 | | 🗸 | | 🗸 | | 🗸 | | *100%* | |  |
|  | *c) Duration of a session (min)* | | 🗸 | | 🗸 | | - | | - | | 🗸 | | 🗸 | | - | | 🗸 | | - | | - | | 🗸 | | - | | - | | - | | *42.8%* | |  |
|  | *d) Intensity* | | 🗸 | | - | | 🗸 | | 🗸 | | 🗸 | | 🗸 | | 🗸 | | 🗸 | | 🗸 | | - | | 🗸 | | 🗸 | | 🗸 | | 🗸 | | *85.7%* | |  |
|  | *e) Type* | | 🗸 | | 🗸 | | 🗸 | | 🗸 | | 🗸 | | 🗸 | | 🗸 | | 🗸 | | 🗸 | | 🗸 | | 🗸 | | 🗸 | | 🗸 | | 🗸 | | *100%* | |  |
|  | *f) Level* | | - | | 🗸 | | - | | - | | - | | - | | - | | - | | 🗸 | | - | | - | | 🗸 | | - | | - | | *21.4%* | |  |
|  | *g) Combination* | | 🗸 | | - | | - | | 🗸 | | 🗸 | | - | | - | | - | | - | | - | | - | | - | | - | | 🗸 | | *28.6%* | |  |
| 9 | Tailoring | | 1 | | 0 | | 0 | | 1 | | 0 | | 1 | | 1 | | 1 | | 0 | | 1 | | 1 | | 1 | | 1 | | 1 | | 71.4% | |  |
| 10 | Modification | | 0 | | 0 | | 0 | | 0 | | 0 | | 0 | | 0 | | 0 | | 0 | | 0 | | 0 | | 0 | | 0 | | 0 | | 0% | |  |
| 11 | Adherence | | 0 | | 0 | | 0 | | 0 | | 0 | | 0 | | 0 | | 0 | | 0 | | 0 | | 0 | | 0 | | 0 | | 0 | | 0% | |  |
| 12 | Delivered | | 0 | | 0 | | 0 | | 0 | | 0 | | 0 | | 0 | | 0 | | 0 | | 0 | | 0 | | 0 | | 0 | | 0 | | 0% | |  |
|  | Total score | | 7 | | 6 | | 3 | | 7 | | 7 | | 8 | | 4 | | 7 | | 4 | | 7 | | 5 | | 4 | | 8 | | 8 | |  | |  |

Each met criteria is 1 point (1: yes; 0: no): 0–4 poor; 5–9 moderate; 10–12 points good. For sub-questions within criteria 8: 🗸: reported; - : not reported.

**S3 Table.** CONTENT scale for therapeutic validity

|  |  |  | Balachandran (2014) | Bean (2009) | Fielding (2002) | Bottaro (2007) | Henwood (2006) | Henwood (2008) | Lopes (2014) | Marsch (2009)  Miszko (2003) | Orr (2006) |  | Reid (2013)  Ramirez-Campillo (2014) |  | Tiggeman (2016) | Zech (2012) | Frequency of reporting | |
| --- | --- | --- | --- | --- | --- | --- | --- | --- | --- | --- | --- | --- | --- | --- | --- | --- | --- | --- |
| 1 | The patient selection is described. | 1 | 1 | 1 | 1 | 1 | 1 | 1 | 1 | 1 | 1 | 1 | 1 | 1 | 1 | 100% | |  |
| 2 | The patient selection is adequate. | 1 | 1 | 1 | 1 | 1 | 1 | 1 | 1 | 1 | 1 | 1 | 1 | 1 | 1 | 100% | |  |
| 3 | Eligibility criteria for therapist and setting determined and adequate. | 0 | 0 | 0 | 0 | 0 | 0 | 0 | 0 | 0 | 0 | 0 | 0 | 0 | 0 | 0% | |  |
| 4 | The therapeutic exercise is based on a-priori aims and intentions. | 1 | 1 | 1 | 1 | 1 | 1 | 1 | 1 | 1 | 1 | 1 | 1 | 1 | 1 | 100% | |  |
| 5 | The rationale for the content and intensity of therapeutic exercise is described and plausible. | 0 | 0 | 0 | 0 | 0 | 0 | 0 | 0 | 0 | 0 | 0 | 0 | 0 | 0 | 0% | |  |
| 6 | The intensity of the therapeutic exercise described. | 1 | 1 | 1 | 1 | 1 | 1 | 1 | 1 | 1 | 1 | 1 | 1 | 1 | 1 | 100% | |  |
| 7 | The therapeutic exercise was monitored and adjusted when considered necessary. | 1 | 1 | 0 | 1 | 1 | 1 | 1 | 1 | 0 | 1 | 1 | 1 | 1 | 0 | 78.6% | |  |
| 8 | The therapeutic exercise was personalized and contextualised to the individual participant. | 0 | 0 | 0 | 0 | 0 | 0 | 0 | 0 | 0 | 0 | 0 | 0 | 0 | 0 | 0% | |  |
| 9 | Adherence to the therapeutic exercise was determined and acceptable. | 0 | 0 | 0 | 0 | 0 | 0 | 0 | 0 | 0 | 0 | 0 | 0 | 0 | 0 | 0% | |  |
|  | Total score | 5 | 5 | 4 | 5 | 5 | 5 | 5 | 5 | 4 | 5 | 5 | 5 | 5 | 4 |  | |  |

Each met criteria is 1 point (1=yes; 0=no). Interventions with a total score >6 are considered therapeutically valid.
